# Comparative study on the immunopotentiator effect of ISA 201, ISA 61, ISA 50, ISA 206 used in trivalent foot and mouth disease vaccine

**DOI:** 10.14202/vetworld.2015.1189-1198

**Published:** 2015-10-15

**Authors:** Ehab El-Sayed Ibrahim, Wael Mossad Gamal, Amr Ismail Hassan, Safy El-Din Mahdy, Akram Zakria Hegazy, Magdy Mahmoud Abdel-Atty

**Affiliations:** Department of Foot and Mouth Disease, Veterinary Serum and Vaccine Research Institute, Abbasia, Cairo, Egypt

**Keywords:** cellular immunity, FMD Montanide ISA vaccines, SNT, ELISA

## Abstract

**Aim:**

A comparison study was conducted to explore the best internationally available adjuvant that could be used in production of a highly potent foot and mouth disease (FMD) vaccine, that could stimulate a strong immune response and possibly give greater protection against FMD.

**Materials and Methods:**

Four experimental batches of trivalent FMD vaccine were prepared with different available oil adjuvants which included Montanide ISA 201, 206, 61 and 50.

**Results:**

The results indicated that vaccines emulsified using Montanide ISA 201 and Montanide ISA 206 adjuvants elicited a protective humoral immune response from the 2^nd^ week postvaccination (WPV) as for ISA 201 with serum neutralization test (SNT) and enzyme-linked immune sorbent assay (ELISA) antibody titers of 1.62±0.047^a^ and 1.8±0.049^a^, 1.59±0.076^a^ and 1.836±0.077^a^, and 1.71±0.06^b^ and 1.96±0.074^b^ for serotypes O, A, SAT2, respectively, and for ISA 206 at SNT and ELISA antibody titers of 1.5±0.082^a^ and 1.84±0.084^a^, 1.56±0.037^a^ and 1.818±0.052^a^, and 1.5±0.106^a,b^ and 1.81±0.104^a,b^ for FMD virus serotypes O, A and SAT2, respectively. For ISA 61 and ISA 50, the protective antibody titer appeared in the 3^rd^ WPV. In the ISA 61 FMD vaccine, SNT and ELISA titer were 1.59±0.076^a^ and 1.9±0.094^a^, 1.53±0.056^a^ and 1.83±0.070^a^, and 1.5±0.082^a^ and 1.84±0.094^a^ for serotypes O, A and SAT2, respectively, and in the case of ISA 50 FMD vaccine, the SNT, and ELISA titer were recorded for serotypes O, A and SAT2 respectively, 1.59±0.037^a^ and 1.8±0.030^a^, 1.68±0.056^a,b^ and 1.916±0.065^a,b^, and 1.65±0.082^a^ and 1.9±0.09^a^. On estimating the cellular immune response, the highest delta optical density levels for ISA 201 (0.395-0.460) and ISA 206 (0.375-0.428) were observed on 14 and 21 days post vaccination (DPV) respectively, while the highest levels of lymphoproliferation for ISA 61 (0.375-0.455) and ISA 50 (0.411-0.430) were on 21 and 28 DPV, respectively.

**Conclusion:**

The duration of immunity from Montanide ISA oils (201, 206, 61 and 50) FMD vaccines is a long-lived immunity which ranged between 32 and 38 weeks post vaccination but the Montanide ISA 201 FMD vaccine is superior to the others in the rapid cellular immune response of the vaccinated animals which showed its highest level within 14 days post vaccination.

## Introduction

Foot and mouth disease (FMD) is an infectious disease of cattle, buffalo, sheep, goats, pigs, and also wild cloven-hoofed animals. FMD virus (FMDV) is the cause of the disease. The virus has seven serological types, identified as; O, A, C, SAT1, SAT2, SAT3 and Asia1 [1,2]. FMD is characterized by fever, lameness and vesicular lesions on the feet, tongue, snout, and teats, with high morbidity and low mortality [3].

Control of FMD through effective vaccination of susceptible animals is considered to be the corner stone to eliminate the disease in endemic areas, but it is considered very difficult as the FMDV is an airborne transmitted virus, contagious nature of the disease [4].

Continuous improvement must be done to the vaccine formulations to obtain highly immunogenic vaccine, and such improvement not only depend on the antigen payload, but also on the adjuvant used in the vaccines so as to protect the susceptible animals in routine and outbreak situations [5].

The oil adjuvant has the capability for generating a rapid, high and long-lasting immune response. Generally, the Montanide series of oil adjuvants (SEPPIC, France) has a clear immunological effect for inactivated vaccine in different susceptible animals [6,7]. The ability to stimulate a serotype specific immune response is an important factor in protecting the livestock from infection. Cell mediated immunity is also important as it can inhibit the subclinical infection in animals, as half of the vaccinated cattle exposed to infection can be a persistently [8,9].

In Egypt, the disease is enzootic and many outbreaks have been reported since 1950. FMD serotypes SAT2, A, and O were last reported in 1950, 1972, and 2000, respectively [10]. The FMDV serotype O was the most prevalent since 1960 and onward [11,12]. FMDV serotype A was reintroduced into Egypt during 2006 through live animal importation where sever clinical signs were recorded among cattle and buffaloes [13]. In addition, serotype SAT2 of FMDV was later introduced into Egypt during 2012, also through the importation of live animals [14]. In Egypt and many other countries, live animal importation is considered as the main risk factor for new outbreaks in Egypt [4]. The in-house produced vaccine by Veterinary Serum and vaccine Research Institute (VSVRI) is the Montanide ISA 206 trivalent inactivated vaccine which consists of three FMDV serotypes (O Pan Asia1, A Iran O5 and SAT2/EGY/2012).

Vaccine adjuvant is very important factor which stimulate specific components of either humeral or cell-mediated immune response [15]. Selecting the ideal or the most suitable adjuvant is one of the important tools in improving the efficacy of the FMD vaccine. An ideal adjuvant is one which can stimulate the humeral immune response early (onset), and promote production of high antibody titers (strength/intensity) that would last long (duration). It should also stimulate the cellular immune response [4,5].

The present study compared different oil based adjuvants that are used in FMD vaccine formulation, on the level of both humoral and cellular immune response. The oil adjuvants were Montanide ISA 201, ISA 61 and ISA 50, and ISA 206.

## Materials and Methods

### Ethical approval

The experiments were carried out according to the protocol of Institutional Animal Ethics Committee and the authors had a permission of the animal owners at the private farms.

### Animals

**Figure-1:**
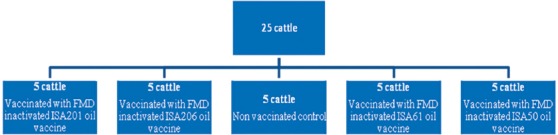
Classification of experimental cattle.

Twenty five apparently healthy native male breed cattle of 1.5 years old of about 300-400 kg body weight from Fayoum farm were used. These cattle were found to be free from antibodies against FMDV serotypes A Iran O5, O Pan Asia1, SAT2/EGY/2012 as screened by serum neutralization test (SNT) and ELISA. The trivalent FMD oil vaccine was inoculated I/M at a dose of 3 ml/animal. A 1 ml dose contain 10^9^ of the FMDV serotypes contained in the vaccine A Iran O5, O Pan Asia1, SAT2/EGY/2012 (Figure-1).

### Suckling baby mice

Fifty suckling Swiss baby mice, 2-4 days old, clinically free from any microbial disease signs (Charles River Strain, USA) were used for the safety of the inactivated antigen.

### Samples

#### Serum samples

A total of 1 and 2 sera samples were collected from the 20 cattles to be vaccinated and 5 un-vaccinated controls prior to vaccination respectively (Day 0). Sera were collected from the cattle every week until 4^th^ weeks, every 2 weeks for 16^th^ weeks, every 4 weeks until 32^nd^ weeks, and lastly, every 2 weeks until the end of the experiment at 42^nd^ weeks.

#### Cell culture

Baby Hamster kidney cell line (BHK_21_) clone 13 obtained from VSVRI, Abbasia, Cairo using Eagl’s medium with 8-10% bovine serum as described by Xuan *et al*. [16] were used for SNT, virus titration and vaccine preparation.

### Viruses

#### FMDV strains

Local FMDV serotypes O Pan Asia1, A Iran O5 and SAT2/EGY/2012 were utilized in the experiments. Which was isolated from field and propagated in BHK_21_ cell line in monolayer cultures for preparation of virus fluid at the Department of FMDs Research, VSVRI, all serotypes were confirmed by world reference laboratory for FMD, Pirbright London, UK.

#### Virus infectivity and antigenicity

The virus titration was carried out by microneutralization in BHK_21_ tissue culture for the three serotypes and the infectivity titer was calculated and expressed in log_10_ 50% tissue culture infectious dose (TCID_50_) as described by Reed and Muench [17] and the complement fixation test was carried out according to Health Protection Agency [18].

#### Virus purification

Aseptically, the harvested culture medium from FMDV infected BHK_21_ cell cultures was centrifuged in a cooling centrifuge at 3000 rpm for 20 min to remove cell debris [19].

#### Virus inactivation

FMDV (O Pan Asia1, A Iran O5, and SAT2/EGY/2012) of the seventh passage on BHK_21_ monolayer with an infectivity titer of 10^8^ TCID_50_/dose. Inactivation was achieved by using a combination of 1 mM binary ethyleneimine (BEI) and 0.04% formaldehyde according to the method described by Barteling and Cassim [20], Ismail *et al*. [21] sodium thiosulfate 20% was added to the virus samples to the inactivated virus to neutralize the BEI in a final concentration of 2%. Sodium bisulfite 20% was added after inactivation process to neutralize the excess of formalin in final concentration of 2%.

### Concentration of the FMDV serotypes

The tissue culture viral fluids of the three serotypes (O Pan Asia1, A Iran O5 and SAT2/EGY/2012) were centrifuged at 7000 revolution/min for 30 min and then concentrated by polyethylene glycol-6000 to reach to 1/10 of their original volumes [19].

### Formulation of the prepared vaccines

The vaccine formulation was done according to Gamil [22]:

- Vaccine 1: Trivalent inactivated FMD with Montanide ISA 201
- Vaccine 2: Trivalent inactivated FMD with Montanide ISA 206
- Vaccine 3: Trivalent inactivated FMD with Montanide ISA 61
- Vaccine 4: Trivalent inactivated FMD with Montanide ISA 50.

### Evaluation of the prepared vaccine formulations

#### Sterility and safety testing

The prepared vaccines were tested for freedom from aerobic and anaerobic bacteria; fungal and mycoplasma contaminants. The vaccines samples were cultured on thioglycolate broth, Sabouraud’s, nutrient agar; phenol dextrose media and mycoplasma medium. The safety of the prepared vaccines was done according to OIE [23], Khushi *et al.*, [24].

### Evaluation of the humeral immune response to the different prepared FMD vaccine

Serum samples collected from the vaccinated and nonvaccinated cattle were tested for the presence of antibody titers against the three serotypes of FMDV (O Pan Asia1, A Iran O5 and SAT2/EGY/2012) by SNT using the technique described by Ferreira [25] and indirect ELISA according to Voller *et al*. [26].

Stimulation of the cellular immune response by the different prepared FMD vaccine was evaluated using the lymphocyte proliferation test according to Lee [27].

### Statistical analysis

Data were analyzed using analysis of variance (ANOVA) in the SPSS-12 statistical software package for P.C.S. Multiple comparisons of means were made using Duncan’s multiple range tests at P< 0.05 %. The results represent the average of five replicates and are presented as the mean ± standard error.

## Results and Discussion

FMD vaccines can be defined as a fixed formulation of defined amount of one or more chemically inactivated cell culture derived preparation of a seed virus strains bended with a suitable adjuvant. Selecting the suitable vaccine formulation is dependent on several factors as the onset of protection, the intensity (titer) of protecting antibody, the duration of protection against FMD.

The vaccine formulations were prepared from three serotypes of FMDV (A IranO5, O Pan Asia, and SAT2/EGY/2012), and it was free from aerobic and anaerobic bacteria; fungal and mycoplasma contaminants. So, the formulated vaccines are safe for animal use.

One of the main factors in selecting the suitable adjuvant is the viscosity of the oil adjuvant used as one of the major drawbacks of the oil adjuvant vaccine is that their use can results in undesirable side effects as granulomas and cysts. The viscosity of the oil adjuvants used in this comparative study (Table-1), as stated by the manufacture (Seppic, Paris) is 30 mPa.s at 25°C for the Montanide ISA 201 and ISA 206 and 35 mPa.s at 25°C for the Montanide ISA 61, but the viscosity for the Montanide ISA 50 is 200 mPa.s at 25°C. It has been suggested that the use of oils used as adjuvants should be of low viscosity as stated by Bomford [28].

**Table-1:** Different types of FMD vaccine formulation used in the study.

Vaccine component	Vaccine 1	Vaccine 2	Vaccine 3	Vaccine 4
Antigen		FMDV (O Pan Asia1, A Iran O5 and SAT2/EGY/2012)		
Adjuvant (montanide)	ISA 201	ISA 206	ISA 61	ISA 50
Type of emulsion	Water-in-oil-in-water	Water-in-oil-in-water	Water-in-oil	Water-in-oil
Oil viscosity at 25°C (mPa.s)	30	30	35	200

FMDV=Foot and mouth disease virus

### Tracing the antibody titer against FMDV serotype (O)

**Figure-2:**
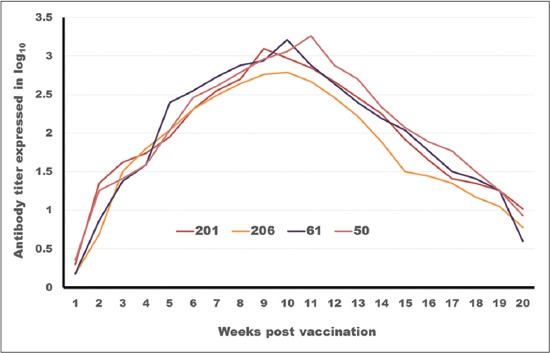
Tracing of antibody titer against foot and mouth disease virus type (O) in different oil vaccines estimated by serum neutralization test.

**Figure-3:**
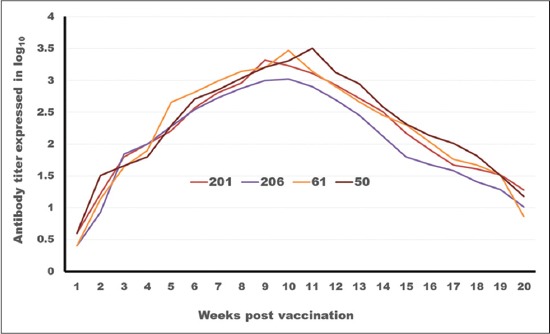
Tracing of antibody titer against foot and mouth disease virus type (O) in different oil vaccines estimated by enzyme-linked immune sorbent assay.

The SNT and ELISA data (Table-2; Figures-2 and -3) show differences in the onset, intensity and duration of the FMD serotype O antibodies elicited by the different vaccine formulations. Concerning the onset of protection, it is clear that ISA 201 protection titer (1.62±0.047^a^ as SNT and 1.8±0.049^a^ as ELISA) and ISA 206 (1.5±0.082^a^ as SNT and 1.84±0.084^a^ as ELISA) appear in the 2^nd^ week post vaccination (WPV) while ISA 61 and ISA 50 appear later in the 3^rd^ WPV (1.59±0.076^a^ as SNT, 1.9±0.094^a^ as ELISA for ISA61 and 1.59±0.037^a^ as SNT, 1.8±0.030^a^ as ELISA for ISA 50). These results came parallel to the results described by Dong *et al*. [29] who mentioned that the ELISA antibodies against FMDV type O were compared between the 2 oil 201, 206. The antibody titer induced by 201-vaccine were higher than which of 206-vaccine on 3dpv, 7dpv, 14dpv, 21dpv, 28dpv. This means that the effect of 201-vaccine in inducing antibody is better than which of 206-vaccine. The protective antibody titer of FMD using SNT is 1.5 log_10_ and by ELISA 1.8 log_10_ according to OIE [23].

**Table-2:** Tracing of antibody titer against FMDV type (O) in different oil vaccines estimated by SNT and ELISA.

Type of vaccine	ISA 201		ISA 206		ISA 61		ISA 50	
				
WPV	Mean SNT±SE	Mean ELISA±SE	Mean SNT±SE	Mean ELISA±SE	Mean SNT±SE	Mean ELISA±SE	Mean SNT±SE	Mean ELISA±SE
0	0.3±0.047^a^	0.6±0.043^a^	0.18±0.088^a^	0.4±0.102^a^	0.18±0.12^a^	0.4±0.19^a^	0.36±0.102^a^	0.6±0.116^a^
1	1.35±0.077^b^	1.22±0.077^b^	0.69±0.09^a^	0.926±0.099^a^	0.87±0.073^a,b^	1.132±0.00^a,b^	1.26±0.037^c^	1.506±0.038^c^
2	1.62±0.047^a^	1.8±0.049^a^	1.5±0.082^a^	1.84±0.084^a^	1.38±0.129^a^	1.64±0.14^a^	1.41±0.037^a^	1.66±0.045^a^
3	1.74±0.037^a^	2±0.045^a^	1.8±0.095^a^	2±0.104^a^	1.59±0.076^a^	1.9±0.094^a^	1.59±0.037^a^	1.8±0.030^a^
4	1.95±0.067^a^	2.21±0.070^a^	2.04±0.076^a^	2.28±0.087^a^	2.4±0.067^b^	2.66±0.076^b^	2.04±0.102^a^	2.29±0.100^a^
6	2.31±0.122^a^	2.57±0.128^a^	2.31±0.06^a^	2.546±0.075^a^	2.55±0.095^c^	2.812±0.097^a^	2.46±0.06^a^	2.706±0.048^a^
8	2.55±0.067^a,b^	2.81±0.075^a,b^	2.49±0.076^a^	2.726±0.088^a^	2.73±0.056^b^	2.992±0.043^b^	2.61±0.09^a,b^	2.856±0.084^a,b^
10	2.7±0.095^a,b^	2.96±0.104^a,b^	2.64±0.06^a^	2.876±0.03^a^	2.88±0.056^b^	3.142±0.043^b^	2.79±0.076^c,b^	3.036±0.084^a,b^
12	3.1±0.037^b^	3.32±0.037^b^	2.76±0.037^a^	2.996±0.043^a^	2.94±0.06^b^	3.202±0.053^b^	2.96±0.056^b^	3.206±0.05^b^
14	2.97±0.073^b^	3.23±0.066^b^	2.79±0.06^a^	3.026±0.0568^a^	3.21±0.037^c^	3.472±0.037^c^	3.06±0.037^b,c^	3.306±0.042^b^
16	2.85±0.082^a^	3.11±0.074^a,b^	2.67±0.10^a^	2.906±0.101^a^	2.88±0.56^a^	3.142±0.043^b^	3.26±0.083^b^	3.506±0.070^c^
20	2.67±0.073^a,b^	2.93±0.066^a,b^	2.46±0.102^a^	2.696±0.095^a^	2.64±0.06^a,b^	2.902±0.052^ab^	2.88±0.06^b^	3.126±0.121^b^
24	2.46±0.076^a,b^	2.72±0.066^b,c^	2.22±0.110^a^	2.456±0.099^a^	2.4±0.082^a^	2.662±0.074^a,b^	2.7±0.095^a^	2.946±0.089^c^
28	2.25±0.142^b^	2.51±0.144^b^	1.89±0.09^a^	2.126±0.088^a^	2.19±0.102^a,b^	2.452±0.093^b^	2.34±0.112^b^	2.586±0.098^b^
32	1.92±0.056^b^	2.17±0.058^b^	1.5±0.095^a^	1.8±0.103^a^	2.04±0.122^b^	2.302±0.118^b^	2.07±0.1^b^	2.316±0.091^b^
34	1.65±0.047^b^	1.91±0.043^b^	1.44±0.076^a^	1.676±0.081^a^	1.77±0.056^b,c^	2.032±0.047^b,c^	1.89±0.076^c^	2.136±0.068^c^
36	1.41±0.076^a^	1.67±0.086^a^	1.35±0.095^a^	1.586±0.103^a^	1.5±0.082^c^	1.762±0.07^a^	1.77±0.073^b^	2.016±0.065^b^
38	1.35±0.082^a,b^	1.61±0.084^a,b^	1.17±0.087^a^	1.406±0.096^a^	1.41±0.102^a,b^	1.672±0.086^b^	1.5±0.047^b^	1.82±0.054^b^
40	1.26±0.076^a^	1.52±0.086^a^	1.05±0.082^a^	1.286±0.093^a^	1.26±0.102^a^	1.522±0.09^a^	1.26±0.076^a^	1.506±0.069^a^
42	1.02±0.073^b^	1.28±0.082^b^	0.78±0.087^a^	1.016±0.092^a,b^	0.6±0.106^a^	0.862±0.107^a^	0.93±0.0561^b^	1.176±0.059^b^

SNT=Serum neutralization test, ELISA=Enzyme linked immune sorbent assay, WPV=Week post vaccination, SE=Standard error, FMDV=Foot and mouth disease virus, different letters indicate significant difference between different treatments at p<0.05 according to Duncan’s multiple range test

### Tracing the antibody titer against FMDV serotype (A)

**Figure-4:**
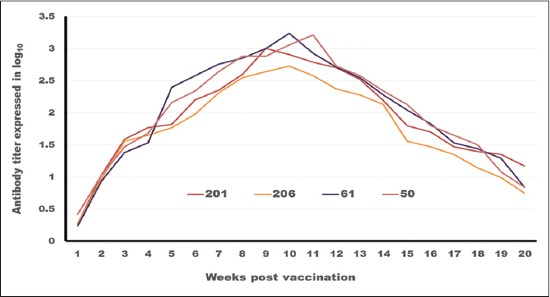
Tracing of antibody titer against foot and mouth disease virus type (A) in different oil vaccines estimated by serum neutralization test.

**Figure-5:**
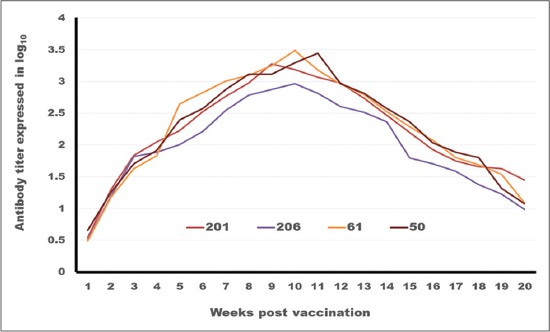
Tracing of antibody titer against foot and mouth disease virus (A) in different oil vaccines estimated by enzyme-linked immune sorbent assay.

Table-3 and Figures-4 and -5 tabulates and depicts FMDV serotype A antibody titers as measured by SNT and ELISA elicited by different vaccine formulations. With regards to the onset of protection, it is clear that ISA 201 and ISA 206 protection titer appear in the 2^nd^ WPV (1.59±0.076^a^ as SNT and 1.836±0.077^a^ as ELISA for ISA 201, 1.56±0.037^a^ as SNT and 1.818±0.052^a^ as ELISA for ISA 206). While ISA 61 and ISA 50 appear later in the 3^rd^ WPV. The protection titer in case ISA 61 were 1.53±0.056^a^ as SNT, 1.83±0.070^a^ as ELISA and 1.68±0.056^a,b^ as SNT, 1.916±0.065^a,b^ as ELISA for ISA 50.

**Table-3:** Tracing of antibody titer against FMDV type (A) in different oil vaccines estimated by SNT and ELISA.

Type of vaccine	ISA 201		ISA 206		ISA 61		ISA 50	
				
WPV	Mean SNT±SE	Mean ELISA±SE	Mean SNT±SE	Mean ELISA±SE	Mean SNT±SE	Mean ELISA±SE	Mean SNT±SE	Mean ELISA±SE
0	0.27±0.073^a^	0.546±0.072^a^	0.27±0.056^a^	0.506±0.073^a^	0.24±0.112^a^	0.488±0.115^a^	0.42±0.129^a^	0.656±0.147^a^
1	1.02±0.110^a^	1.296±0.112^a^	0.96±0.037^a^	1.196±0.046^a^	0.93±0.12^a^	1.178±0.131^a^	1.02±0.129^a^	1.256±0.140^a^
2	1.59±0.076^a^	1.836±0.077^a^	1.56±0.037^a^	1.818±0.052^a^	1.38±0.073^a^	1.628±0.075^a^	1.47±0.056^a^	1.706±0.070^a^
3	1.77±0.056^b^	2.046±0.052^b^	1.65±0.025^a,b^	1.886±0.018^a,b^	1.53±0.056^a^	1.83±0.070^a^	1.68±0.056^a,b^	1.916±0.065^a,b^
4	1.82±0.047^b^	2.226±0.047^b^	1.77±0.03^a^	2.006±0.016^a^	2.4±0.047^d^	2.648±0.052^d^	2.16±0.037^c^	2.396±0.025^c^
6	2.2±0.067^b^	2.526±0.067^b^	1.98±0.073^a^	2.216±0.084^a^	2.58±0.056^c^	2.828±0.073^c^	2.34±0.037^b^	2.576±0.040^b^
8	2.35±0.06^b^	2.766±0.057^b^	2.31±0.06^a^	2.546±0.065^a^	2.76±0.06^c^	3.008±0.072^c^	2.64±0.06^b,c^	2.876±0.049^b,c^
10	2.6±0.047^b^	2.976±0.040^b^	2.55±0.047^a^	2.786±0.061^a^	2.85±0.047^c^	3.098±0.052^b^	2.88±0.056^c^	3.116±0.06^b^
12	3±0.095^b^	3.276±0.086^b^	2.64±0.037^a^	2.876±0.041^a^	3±0.047^b^	3.248±0.065^b^	2.88±0.056^b^	3.116±0.06^b^
14	2.91±0.037^b^	3.186±0.029^b^	2.73±0.056^a^	2.966±0.065^a^	3.24±0.09^c^	3.488±0.107^c^	3.06±0.037^b^	3.296±0.039^b,c^
16	2.79±0.06^a,b^	3.066±0.052^b^	2.58±0.056^a^	2.816±0.065^a^	2.93±0.07^b^	3.178±0.083^b^	3.21±0.102^c^	3.446±0.1^c^
20	2.7±0.047^b^	2.976±0.045^b^	2.37±0.03^a^	2.606±0.038^a^	2.71±0.062^b^	2.958±0.055^b^	2.73±0.073^b^	2.966±0.056^b^
24	2.52±0.06^a^	2.736±0.055^a,b^	2.28±0.08746^a^	2.516±0.0871^a^	2.55±0.125^a^	2.798±0.125^b^	2.58±0.087^a^	2.816±0.073^b^
28	2.19±0.139^a^	2.466±0.142^a^	2.13±0.056^a^	2.366±0.048^a^	2.28±0.12^a^	2.528±0.121^a^	2.34±0.076^a^	2.576±0.0762^a^
32	1.8±0.056^b^	2.196±0.06^b^	1.56±0.076^a^	1.796±0.070^a^	2.04±0.147^b^	2.288±0.143^b^	2.13±0.073^b^	2.366±0.072^b^
34	1.7±0.047^a,b^	1.926±0.050^b^	1.47±0.03^a^	1.706±0.038^a^	1.83±0.087^b^	2.078±0.089^b^	1.8±0.067^b^	2.036±0.074^b^
36	1.47±0.056^a,b^	1.746±0.055^a,b^	1.35±0.047^a^	1.586±0.048^a^	1.53±0.056^b,c^	1.8±0.062^b^	1.65±0.067^c^	1.886±0.074^b^
38	1.4±0.056^b^	1.656±0.055^b^	1.14±0.076^a^	1.376±0.085^a^	1.44±0.037^b^	1.688±0.046^b^	1.5±0.082^b^	1.8±0.08262^b^
40	1.35±0.067^c^	1.626±0.063^c^	0.99±0.09^a^	1.226±0.090^a^	1.29±0.06^c^	1.538±0.074^b,c^	1.08±0.087^a,b^	1.316±0.09791^a,b^
42	1.17±0.073^b^	1.446±0.07^b^	0.75±0.082^a^	0.986±0.079^a^	0.84±0.076^a^	1.088±0.069^a^	0.84±0.112^a^	1.076±0.11338^a^

SNT=Serum neutralization test, ELISA=Enzyme linked immune sorbent assay, WPV=Week post vaccination, SE=Standard error, FMDV=Foot and mouth disease virus, different letters indicate significant difference between different treatments at p<0.05 according to Duncan’s multiple range test

### Tracing the antibody titer against FMDV serotype (SAT2)

**Figure-6:**
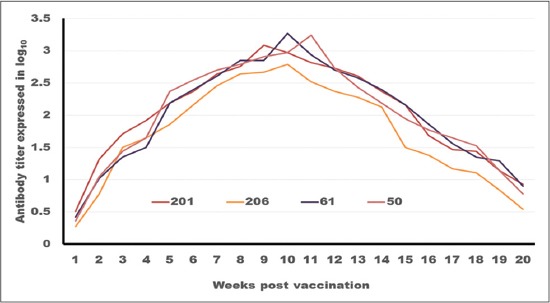
Tracing of antibody titer against foot and mouth disease virus type (SAT2) in different oil vaccines estimated by serum neutralization test.

**Figure-7:**
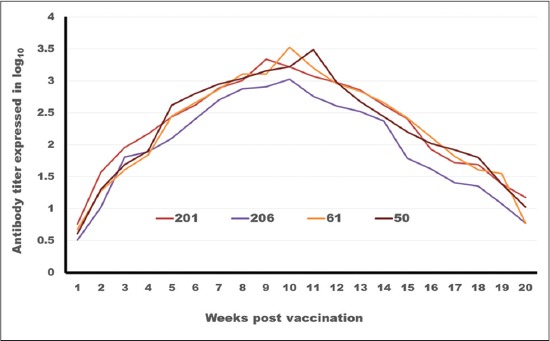
Tracing of antibody titer against foot and mouth disease virus type (SAT2) in different oil vaccines estimated by enzyme-linked immune sorbent assay.

On analyzing the data in Table-4 and Figures-6 and -7 which trace the antibody titer against FMDV serotype SAT2.

**Table-4:** Tracing of antibody titer against FMDV type (SAT2) in different oil vaccines estimated by SNT and ELISA.

Vaccine type	ISA 201		ISA 206		ISA 61		ISA 50	
				
WPV	Mean SNT±SE	Mean ELISA±SE	Mean SNT±SE	Mean ELISA±SE	Mean SNT±SE	Mean ELISA±SE	Mean SNT±SE	Mean ELISA±SE
0	0.51±0.06^a^	0.76±0.077^a^	0.27±0.056^a^	0.51±0.074^a^	0.42±0.129^a^	0.68±0.139^a^	0.36±0.122^a^	0.61±0.139^a^
1	1.32±0.087^b^	1.57±0.1^b^	0.78±0.087^a^	1.02±0.098^a^	1.02±0.129^a,b^	1.28±0.141^a,b^	1.05±0.067^a,b^	1.3±0.081^a,b^
2	1.71±0.06^b^	1.96±0.074^b^	1.5±0.106^a,b^	1.81±0.104^a,b^	1.35±0.082^a^	1.61±0.095^a^	1.44±0.076^a^	1.69±0.091^a,b^
3	1.92±0.056^b^	2.17±0.68^b^	1.65±0.067^a^	1.89±0.06^a^	1.5±0.082^a^	1.84±0.094^a^	1.65±0.082^a^	1.9±0.09^a^
4	2.19±0.06^b^	2.44±0.04^b^	1.86±0.102^a^	2.1±0.109	2.19±0.037^b^	2.45±0.036^b^	2.37±0.073^b^	2.62±0.074^b^
6	2.37±0.056^a,b^	2.62±0.066^a,b^	2.16±0.112^a^	2.4±0.116^a^	2.4±0.047^a,b^	2.66±0.042^b^	2.55±0.082^b^	2.8±0.083^b^
8	2.64±0.06^a^	2.89±0.075^a^	2.46±0.139^a^	2.7±0.144^a^	2.61±0.037^a^	2.87±0.027^a^	2.7±0.047^a^	2.95±0.053^a^
10	2.76±0.076^a,b^	3.01±0.091^a,b^	2.64±0.076^a^	2.88±0.072^a^	2.85±0.047^b^	3.11±0.05^b^	2.79±0.037^a,b^	3.04±0.041^a,b^
12	3.09±0.076^c^	3.34±0.086^c^	2.67±0.056^a^	2.91±0.045^a^	2.85±0.047^a,b^	3.11±0.05^b^	2.91±0.06^b,c^	3.16±0.062^b,c^
14	2.97±0.03^a^	3.22±0.035^a^	2.79±0.09^a^	3.03±0.082^a^	3.27±0.087^b^	3.53±0.088^b^	2.97±0.056^a^	3.22±0.059^a^
16	2.82±0.03^b^	3.07±0.035^b^	2.52±0.073^a^	2.76±0.058^a^	2.94±0.102^b^	3.2±0.105^b^	3.24±0.09^c^	3.49±0.101^c^
20	2.73±0.056^b^	2.98±0.056^b^	2.37±0.073^a^	2.61±0.058^a^	2.7±0.067^b^	2.96±0.06^b^	2.73±0.087^b^	2.98±0.094^b^
24	2.61±0.06^b^	2.86±0.059^b^	2.28±0.1^a^	2.52±0.09^a^	2.58±0.087^b^	2.84±0.082^b^	2.43±0.110^a,b^	2.68±0.104^a,b^
28	2.37±0.03^b^	2.62±0.035^b,c^	2.13±0.073^a^	2.37±0.067^a^	2.4±0.047^b^	2.66±0.036^c^	2.19±0.102^a,b^	2.44±0.102^a,b^
32	2.16±0.037^c^	2.41±0.041^c^	1.5±0.047^a^	1.79±0.064^a^	2.16±0.06^c^	2.42±0.050^c^	1.95±0.082^b^	2.2±0.075^b^
34	1.68±0.056^b^	1.93±0.068^b^	1.381±0.056^a^	1.62±0.071^a^	1.86±0.06^c^	2.12±0.047^c^	1.77±0.056^b,c^	2.02±0.049^b,c^
36	1.47±0.073^b^	1.72±0.079^b^	1.17±0.073^a^	1.41±0.089^a^	1.56±0.037^b^	1.82±0.034^b^	1.65±0.047^b^	1.92±0.047^b^
38	1.44±0.06^b^	1.69±0.063^b^	1.11±0.112^a^	1.35±0.128^a^	1.35±0.067^b^	1.61±0.053^b^	1.53±0.056^b^	1.8±0.059^b^
40	1.14±0.09^b^	1.39±0.092^b^	0.84±0.06^a^	1.08±0.072^a^	1.29±0.102^b^	1.55±0.09^b^	1.14±0.102^b^	1.39±0.11^b^
42	0.93±0.137^a^	1.18±0.139^b^	0.54±0.102^a^	0.78±0.114^a^	0.9±0.157^a^	0.78±0.114^a^	0.78±0.087^a^	1.03±0.076^a,b^

SNT=Serum neutralization test, ELISA=Enzyme linked immune sorbent assay, WPV=Week post vaccination, SE=Standard error, FMDV=Foot and mouth disease virus, different letters indicate significant difference between different treatments at p<0.05 according to Duncan’s multiple range test

Concerning the onset of protection, it is clear that ISA 201 and ISA 206 protection titer appear in the 2^nd^ WPV, but there is statistically significant difference in the antibody titer (intensity) as ISA 201 (1.71±0.06^b^ as SNT and 1.96±0.074^b^ as ELISA) gave more titer than ISA 206 (1.5±0.106^a,b^ as SNT and 1.81±0.104^a,b^ as ELISA) while ISA 61 and ISA 50 appear later in the 3^rd^ WPV (1.5±0.082^a^ as SNT and 1.84±0.094^a^ as ELISA for ISA 61 and 1.65±0.082^a^ as SNT and 1.9±0.09^a^ as ELISA for ISA 50). These results may be attributed to the mode of action of oil adjuvants which delaying the absorption of antigen and stimulate mononuclear cells to produce antibodies at local and distant sites so elicit both humoral and cellular immunity [30].

The SNT and ELISA antibody titer against FMDV serotype O, A and SAT2 are negative along the whole experiment (42 weeks) in the non vaccinated control cattle.

From Tables-2-5 concerning the duration of protective immunity for FMD serotypes (O Pan Asia1, A Iran O5, and SAT2/EGY/2012), there were a significant difference among the different types of oil adjuvants used, where the protective antibodies were detected until 32^nd^, 34^th^, 36^th^_,_ and 38^th^ WPV for ISA 206, ISA 201, ISA 61, and ISA 50, respectively. These results were agreement with Fakhry *et al*. [31] who concluded that the FMD duration of immunity elicited by Montanide oils ISA 206 and ISA 50 reach 32 and 38 WPV respectively.

**Table-5:** Summary for the previous results of the onset and duration of immunity in vaccinated calves with inactivated trivalent FMD vaccine with different oil adjuvants against serotypes (O, A and SAT2).

Type of vaccine	Onset at week	SNT antibody titer			ELISA antibody titer			Peak at week	SNT antibody titer			ELISA antibody titer			Duration of immunity
			
		O	A	SAT2	O	A	SAT2		O	A	SAT2	O	A	SAT2	
Montanide ISA 201 oil vaccine	2^nd^	1.62	1.59	1.71	1.8	1.836	1.96	12^th^	3.1	3	3.09	3.32	3.276	3.34	34
Montanide ISA 206 oil vaccine	2^nd^	1.5	1.56	1.5	1.84	1.818	1.81	14^th^	2.79	2.73	2.79	3.026	2.966	3.03	32
Montanide ISA 61 oil vaccine	3^rd^	1.59	1.53	1.5	1.9	1.83	1.84	14^th^	3.21	3.24	3.27	3.472	3.488	3.53	36
Montanide ISA 50 oil vaccine	3^rd^	1.59	1.68	1.65	1.8	1.916	1.9	16^th^	3.26	3.21	3.24	3.506	3.446	3.49	38

FMD=Foot and mouth disease, SNT=Serum neutralization test, ELISA=Enzyme linked immune sorbent assay

Cell mediated immunity is a component of an animal’s immune response to FMDV infection and vaccination. Stimulation of cell mediated immune response was evaluated using the lymphocytic proliferation assay, expressed as delta optical density (ΔOD), for all the vaccine groups [32,33].

The ΔOD of all vaccinated groups were demonstrated from the 3^rd^ till the 35^th^ day post vaccination (DPV) (Table-6). The ΔOD of the nonspecific mitogen, phytohemagglutinin, and specific mitogens, FMDV (as) on the third DPV were 0.275-0.365, 0.245-0.295, 0.255-0.335 and 0.220-0.265 for Montanide ISA 201, 206, 61 and 50 respectively.

**Table-6:** Cellular immune response expressed as ΔOD of cattle vaccinated with different oil adjuvanated trivalent FMD vaccines.

Oil adjuvant	Mitogen and used virus	Percentage of lymphocyte proliferation/days post vaccination					

		3^rd^	7^th^	14^th^	21^th^	28^th^	35^th^
Montanide ISA 201 oil vaccine	PHA	0.275	0.311	0.395	0.344	0.285	0.214
	FMDV	0.365	0.438	0.460	0.441	0.351	0.272
Montanide ISA 206 oil vaccine	PHA	0.245	0.310	0.344	0.375	0.295	0.211
	FMDV	0.295	0.315	0.395	0.428	0.321	0.251
Montanide ISA 61 oil vaccine	PHA	0.255	0.310	0.335	0.375	0.260	0.219
	FMDV	0.335	0.395	0.435	0.455	0.335	0.291
Montanide ISA 50 oil vaccine	PHA	0.220	0.295	0.341	0.365	0.411	0.295
	FMDV	0.265	0.314	0.364	0.396	0.430	0.323

FMDV=Foot and mouth disease virus, PHA=Phytohemagglutinin, ΔOD=Delta optical density,

The highest ΔOD levels for ISA 201 (0.395-0.460) and ISA 206 (0.375-0.428) were observed on DPV 14 and 21, respectively, while the highest levels of lymphoproliferation for Montanide ISA 61 (0.375-0.455) and ISA 50 (0.411-0.430) were on DPV 21 and 28 accordingly. These results were partly in agreement with El-Watany *et al*. [34], Mansour [35], Samir [36], El-Din *et al*. [37] in that FMD vaccine stimulated the cellular immune response, and lymphocyte stimulation by FMDV was greater than by mitogens (phytohemagglutinin) and increased on 14 DPV. Moreover, the cellular immune response results clearly indicate that Montanide ISA 201 showed its highest levels on 14 DPV, followed by Montanide ISA 206 and ISA 61 on 21 DPV and ISA 50 on DPV 28.

## Conclusion

It was clear that the duration of immunity from Montanide oils (201, 206, 61 and 50) FMD vaccines is a long-lived immunity which ranged between 32 and 38 weeks post vaccination, but the Montanide ISA 201 FMD vaccine is superior to the others in the rapid cellular immune response of the vaccinated animals which showed its highest level within 2 weeks. Further, studies are recommended to evaluate the effect of these Montanide oils on other cellular immune components such as the interferon, interleukin, and IG2a.

## Authors’ Contributions

EEI: Preparation of BHK21 cell culture, Titration of FMDV serotype O Pan Asia, Formulation to trivalent FMD Montanide ISA 201 oil vaccine, apply SNT and ELISA on the serum samples, make statistical analysis, write the manuscript and follow up the publication process, the corresponding author for research.

WMG: Inoculation of TC with FMDV serotype A IranO5 and sharing in titeration and inactivation process for FMDV serotype A IranO5, Formulation to trivalent FMD Montanide ISA 206 oil vaccine and apply SNT and ELISA on the serum samples and write the manuscript and sharing in publication.

AIH: Inoculation of TC with FMDV serotype SAT2/EGY/2012 and sharing in titeration and inactivation process for FMDV serotype SAT2/EGY/2012, Formulation to trivalent FMD Montanide ISA 50 oil vaccine and apply SNT and ELISA on the serum samples.

SEM: Inoculation of TC with FMDV serotype O Pan Asia and sharing in titeration and inactivation process for FMDV serotype O Pan Asia, Formulation to trivalent FMD Montanide ISA 61 oil vaccine and apply SNT and ELISA on the serum samples.

AZH: Preparation of BHK21 cell culture, Formulation to trivalent FMD Montanide ISA 201 oil vaccine, vaccinated all groups with different formulated vaccines, collection to all sera samples, apply SNT and ELISA on the serum samples

MMA Helped in write the manuscript and follow up all the procedure of preparation and evaluation of vaccines. All authors read and approved the final manuscript.
